# Supplementary material for: Recombinant Fsh and Lh therapy for spawning induction of previtellogenic and early spermatogenic arrested teleost, the flathead grey mullet (Mugil cephalus)
Source: Sci Rep. 2022 Apr 21;12:6563. doi: 10.1038/s41598-022-10371-0 (PMC9023507; doi:10.1038/s41598-022-10371-0)
Supplement: Supplementary file 1 — Supplementary Figures. [file 41598_2022_10371_MOESM1_ESM.pdf]

Supplementary Figures for

Recombinant Fsh and Lh therapy for spawning induction of previtellogenic and early spermatogenic arrested teleost, the flathead grey mullet (*Mugil cephalus*)

Ramos-Júdez, S., Giménez, I., Gumbau-Pous, J., Arnold-Cruañes, L.S., Estévez, A. and Duncan, N.

Sandra Ramos-Júdez, E-mail: sandra.ramos@s2aquacolab.pt

Ignacio Giménez, E-mail: igimenez@raraavis-bio.com

Neil Duncan, E-mail: neil.duncan@irta.cat

This PDF file includes: Figs. S1 to S7

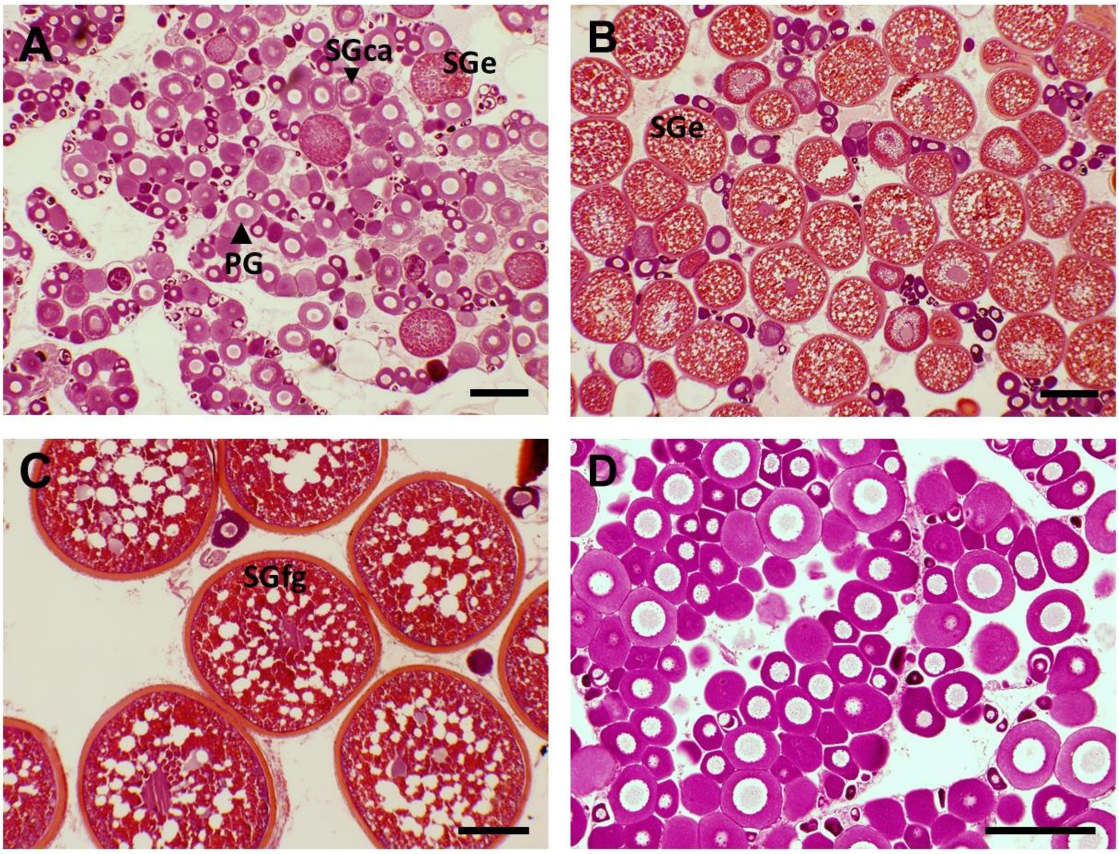

Figure S1. Histological photographs of representative ovarian biopsies from flathead grey mullet females (*Mugil cephalus*) during the experimental period. (**A**) A female in early-vitellogenesis (SGe) with mainly primary growth oocytes, some cortical alveoli stage (SGca) and only a small percentage of oocytes in SGe at the beginning of the experiment, (**B**) a female in SGe after rFsh treatment with a clear clutch of oocytes recruited into vitellogenesis, (**C**) a rGth-treated female with full-grown oocytes (SGfg), and (**D**) a control female in previtellogenesis by the end of the experimental period. Scale bar: 200 μm.

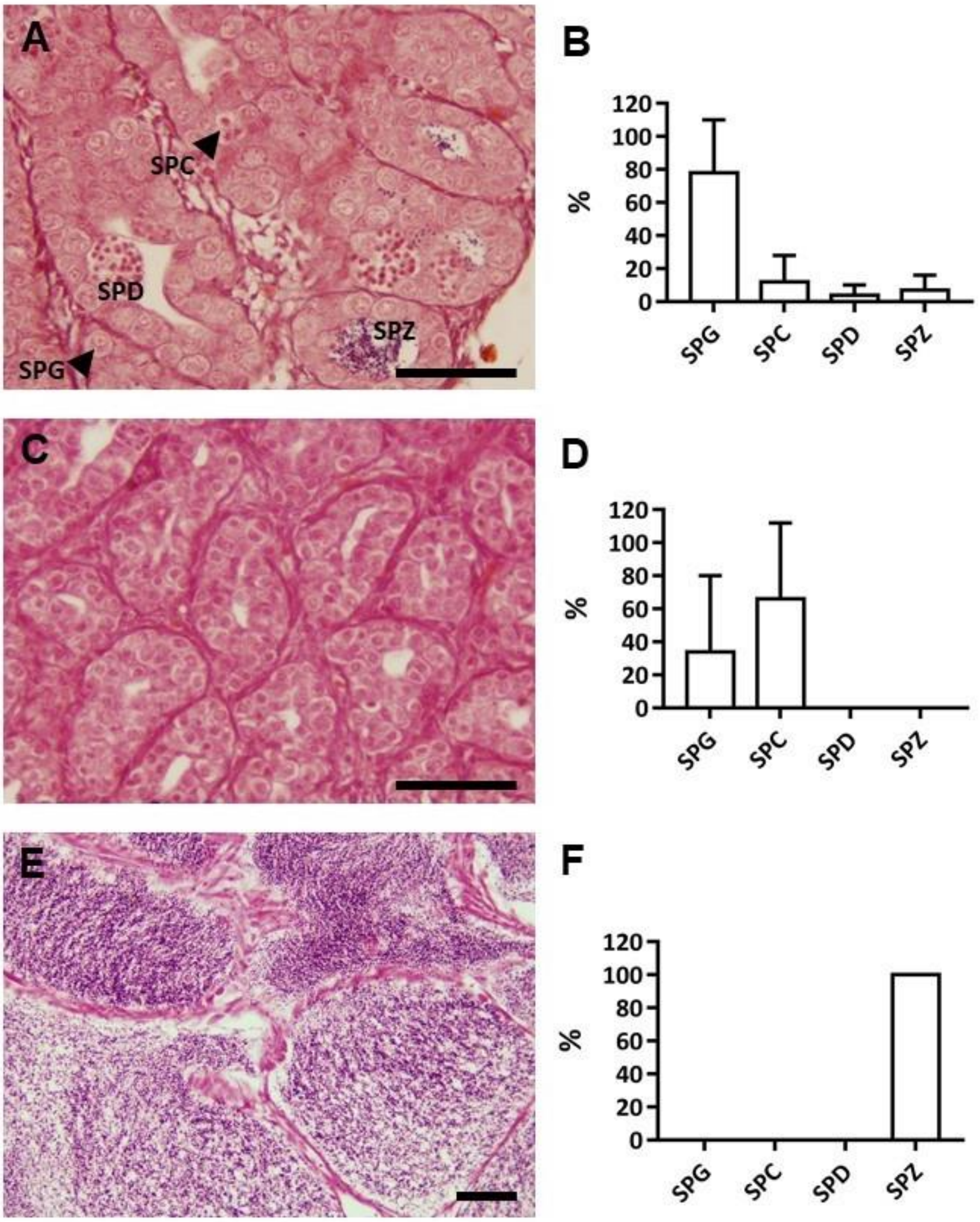

Figure S2. Histological sections of testis and the percentages of the spermatogenesis developmental stages at the beginning of the study (**A** and **B**, n = 2) and at the end of the experimental period in control group (**C** and **D**, n = 2) and rGth-treated group (**E** and **F**, n = 2). SPG, Spermatogonia; SPC, Spermatocyte; SPD, Spermatid; SPZ, Spermatozoa. Scale bars = 50 μm.

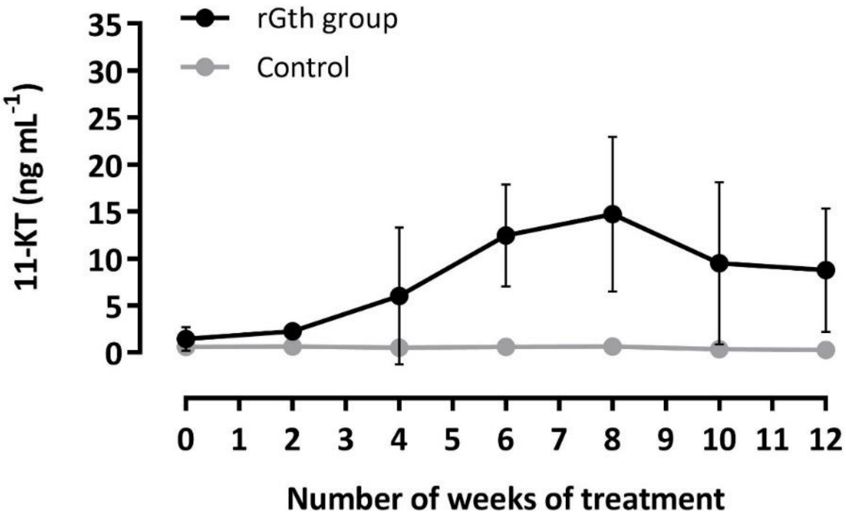

Figure S3. Effect of rFsh and rLh treatment (rGth group) and saline (control) on 11-ketotestosterone (11-KT) levels (mean ± SD) in the flathead grey mullet (*Mugil cephalus*). Data from Group 1 (n = 4 rGth-treated males, n = 3 control) from the first application (week 0) to the last checking (week 12) was combined with data from Group 2 (n = 5 rGth-treated males, n = 3 control) from week 0 to 10 in order to present the results. Two-way RM ANOVA showed a significant effect of the rGths treatment on the production of 11-KT ($P < 0.001$).

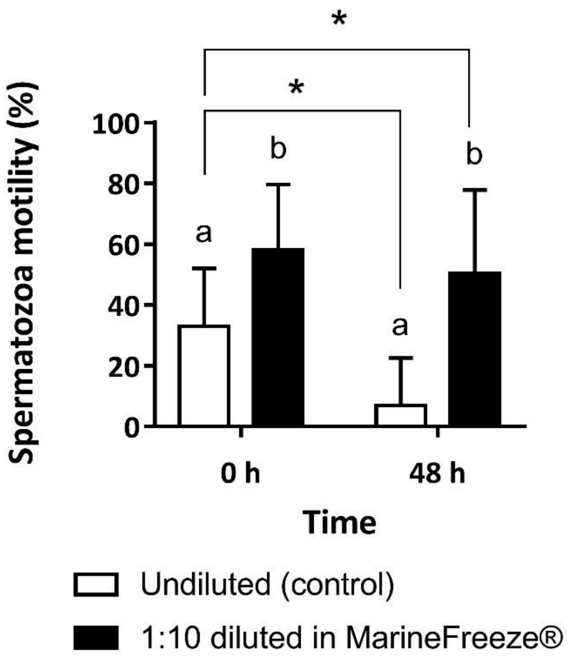

Figure S4. Percentage motility of sperm samples collected from rGth-treated males at the end of the experimental period (week 10 and 13). Two-way RM ANOVA was performed followed by the Holm-Sidak post hoc test with males as subjects, time of storage (0 or 48h) and sample dilution (undiluted or 1:10 diluted in Marine Freeze®) as the independent variables, and percentage motility as the dependent variables. Different letters indicate significant differences between undiluted and diluted samples within the same time of evaluation, while asterisks show significant differences between evaluation time.

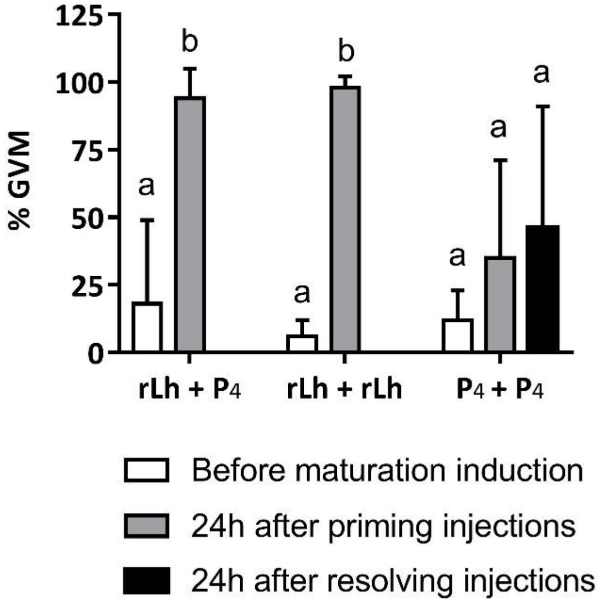

Figure S5. Percentage of oocytes at maturation stage (OM) with germinal vesicle migration (GVM) before OM induction, 24 h after the priming injections and 24 h after the resolving injections. Treatments applied to induce OM and spawning were (i) priming 30 µg kg^{-1} rLh and 40 mg kg^{-1} resolving Progesterone (P_4), (ii) 30 µg kg^{-1} rLh as priming and resolving injections or (iii) 40 mg kg^{-1} P_4 as priming and resolving injections given 24:05 ± 0:40 h apart. Different letters indicate significant differences at different timing of the inductions within a same treatment following one-way RM ANOVA.

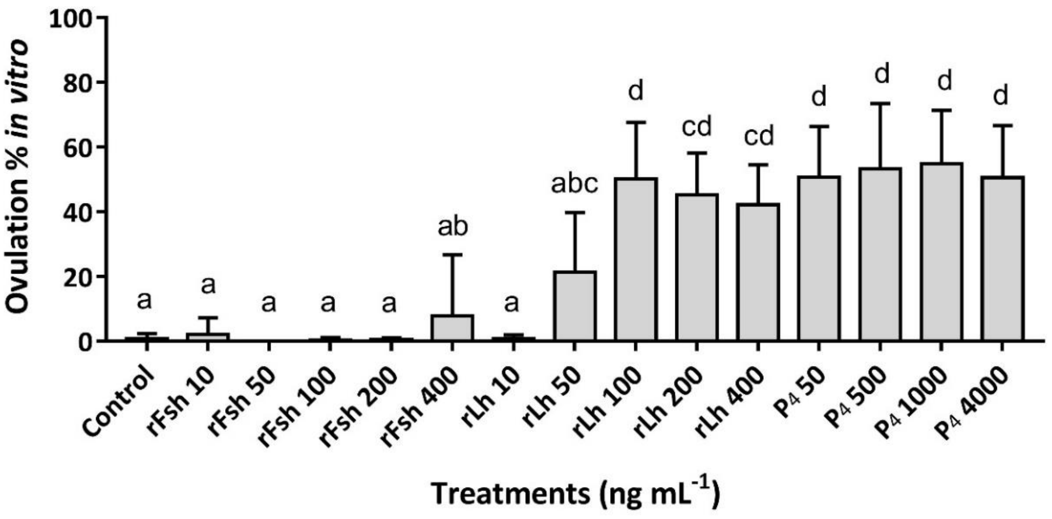

Figure S6. *In vitro* percentage of ovulation (mean ± SD) of oocytes in OM with different effectors, doses and combinations of efectors and doses. Statistical differences between treatments were examined by a one-way repeated measures ANOVA with individual females as subjects (3 replicates per individual, 6 individuals n = 18 wells per hormone concentration).

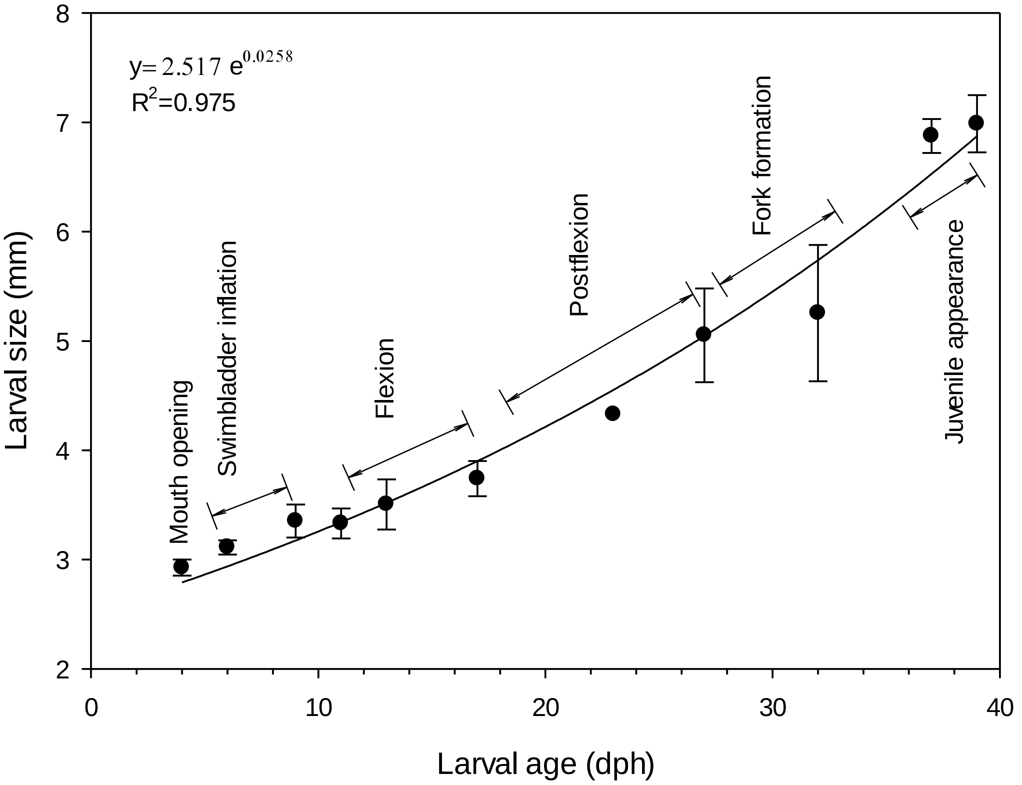

Figure S7. Growth performance of flathead grey mullet (*Mugil cephalus*) larvae in terms of total length (mm, mean ± SD), in relation to time (days post hatch, dph) showing the timing of appearance of the main developmental stages.
